# Linearized Siderophore Products Secreted via MacAB Efflux Pump Protect Salmonella enterica Serovar Typhimurium from Oxidative Stress

**DOI:** 10.1128/mBio.00528-20

**Published:** 2020-05-05

**Authors:** L. M. Bogomolnaya, R. Tilvawala, J. R. Elfenbein, J. D. Cirillo, H. L. Andrews-Polymenis

**Affiliations:** a Texas A&M University Health Science Center, Bryan, Texas, USA; b Institute of Fundamental Medicine and Biology, Kazan Federal (Volga Region) University, Kazan, Russia; c Marshall University Joan C. Edwards School of Medicine, Huntington, West Virginia, USA; d University of Massachusetts Medical School, Worcester, Massachusetts, USA; e North Carolina State University College of Veterinary Medicine, Raleigh, North Carolina, USA; f University of Wisconsin School of Veterinary Medicine, Madison, Wisconsin, USA; University of Michigan-Ann Arbor

**Keywords:** MacAB, *Salmonella*, multidrug efflux pump, siderophores

## Abstract

Nontyphoidal salmonellae (NTS) are exposed to reactive oxygen species (ROS) during their residency in the gut. To survive oxidative stress encountered during infection, salmonellae employ several mechanisms. One of these mechanisms involves the multidrug efflux pump MacAB, although the natural substrate of this pump has not been identified. MacAB homologs in pseudomonads secrete products of nonribosomal peptide synthesis (NRPS). In Salmonella enterica serovar Typhimurium, the siderophore enterobactin is produced by NRPS in response to iron starvation and this molecule can be processed into salmochelin and several linear metabolites. We found that *Salmonella* mutants lacking the key NRPS enzyme EntF are sensitive to peroxide mediated killing and cannot detoxify extracellular H_2_O_2_. Moreover, EntF and MacAB function in a common pathway to promote survival of *Salmonella* during oxidative stress. We further demonstrated that *S.* Typhimurium secretes siderophores in iron-rich media when peroxide is present and that these MacAB-secreted metabolites participate in protection of bacteria against H_2_O_2_. We showed that secretion of anti-H_2_O_2_ molecules is independent of the presence of the known siderophore efflux pumps EntS and IroC, well-described efflux systems involved in secretion of enterobactin and salmochelin. Both salmochelin and enterobactin are dispensable for *S.* Typhimurium protection against ROS; however, linear metabolites of enterobactin produced by esterases IroE and Fes are needed for bacterial survival in peroxide-containing media. We determined that linearized enterobactin trimer protects *S.* Typhimurium against peroxide-mediated killing in a MacAB-dependent fashion. Thus, we suggest that linearized enterobactin trimer is a natural substrate of MacAB and that its purpose is to detoxify extracellular reactive oxygen species.

## Abstract (precis)

Nontyphoidal *Salmonella* bacteria induce a classic inflammatory diarrhea by eliciting a large influx of neutrophils, producing a robust oxidative burst. Despite substantial progress understanding the benefits to the host of the inflammatory response to *Salmonella*, little is known regarding how *Salmonella* can simultaneously resist the damaging effects of the oxidative burst. The multidrug efflux pump *MacAB* is important for survival of oxidative stress both *in vitro* and during infection. We describe a new pathway used by *Salmonella* Typhimurium to detoxify extracellular reactive oxygen species using a multidrug efflux pump (MacAB) to secrete a linear siderophore, a metabolite of enterobactin. The natural substrates of many multidrug efflux pumps are unknown, and functional roles of the linear metabolites of enterobactin are unknown. We bring two novel discoveries together to highlight an important mechanism used by *Salmonella* to survive under the oxidative stress conditions that this organism encounters during the classic inflammatory diarrhea that it also induces.

## INTRODUCTION

Salmonellae are the most common cause of bacterial foodborne disease in the United States and cause hundreds of millions of cases of foodborne disease, invasive nontyphoidal salmonellosis (iNTS), and enteric fever worldwide each year. Nontyphoidal salmonellae (NTS) are the cause of approximately 1.4 million cases/year of gastrointestinal disease in the United States, in the form of a classic inflammatory diarrhea. This inflammatory diarrhea is characterized by an abundance of neutrophils, elicited by CXC chemokines secreted in response to secretion of effectors of type III secretion system 1 (T3SS1) from NTS (1, 2). Thus, NTS must survive the arsenal of bactericidal mechanisms of neutrophils during the intestinal stage of salmonellosis. During systemic infection, *Salmonella* can be found in lesions in the liver and spleen. Inflammatory cells, including neutrophils and macrophages, are present in these lesions in abundance, and *Salmonella* replicate inside macrophages in a “*Salmonella*-containing vacuole” (SCV) (3, 4).

Salmonellae encode at least 11 multidrug efflux systems (ES) with partially characterized specificity for a number of antibiotics and biocides (5, 6). One of these, the ABC-type multidrug efflux system encoded by MacAB, is widely distributed among bacterial species (7, 8). MacAB systems are tripartite efflux systems (TES) that have been previously shown to be involved in the efflux of antibiotics, including macrolides (9). Although the structure and mechanism by which the MacAB pump functions have been carefully examined previously (10, 11), very few natural substrates of MacAB have been identified (heat-stable enterotoxin II and protoporphyrin PPIX [12, 13]). Deletion of the MacAB efflux system in Salmonella enterica serovar Typhimurium renders these organisms avirulent in BALB/c mice, suggesting that this efflux system plays an essential role in virulence (14). Further dissection of the role of MacAB during *Salmonella* infection suggests that this pump participates in the detoxification of extracellular peroxide and in survival of *S.* Typhimurium during exposure to reactive oxygen species (ROS) (15). We presume that the natural substrate of MacAB in *Salmonella* is required for resistance to oxidative stress, but the nature of this substrate has not yet been determined.

Reactive oxygen species generated by NADPH membrane oxidase complex 2 (Nox2) represent a key defense deployed by innate immune cells against bacterial infection. This enzyme complex generates reactive oxygen species that are released from phagocytes both into the extracellular environment and into phagosomes. Hypochlorous acid, superoxide, and hydrogen peroxide are all produced by phagocytes and function to kill microorganisms, although their mechanisms of killing have not been completely elucidated (16, 17). Hydrogen peroxide, produced from superoxide, can cross bacterial membranes and is bactericidal. In the Fenton reaction, H_2_O_2_ reacts with intracellular iron sulfur clusters to produce damaging free radicals, including (among others) hydroxyl radicals (18), that can damage cellular components, including proteins, lipids, and DNA.

Bacterial pathogens possess numerous mechanisms to avoid and overcome the damaging effects of ROS. These mechanisms include direct detoxification of ROS by enzymes, including superoxide dismutases (SODs), catalases, and peroxidases. *S.* Typhimurium expresses cytosolic superoxide dismutases SodA and SodB, also present in Escherichia coli, as well as two periplasmic copper-zinc SODs, SodCI and SodCII (19, 20). SODs convert superoxide anion into H_2_O_2_. Enzymatic detoxification of hydrogen peroxide, in turn, depends on the activity of catalases and peroxidases. *S.* Typhimurium encodes three catalase enzymes, KatE, KatG, and KatN, and two peroxidases, AhpC and TsaA. These enzymes are highly redundant in their ability to degrade intracellular peroxide (21, 22). In addition, catecholate siderophores, metabolites produced by bacteria in response to iron limitation, were recently reported to play a role in protection of *S.* Typhimurium from oxidative stress (23). In previous studies, E. coli mutants lacking *entE*, a subunit of enterobactin synthase, were more sensitive to oxidative killing, leading to the conclusion that enterobactin is required for protection from ROS (24, 25).

Here, we show that the antibiotic efflux system encoded by *macAB* is required for optimal *S.* Typhimurium colonization in the inflamed intestine and we identify the natural substrate of this efflux system. We show that mutants lacking *entF*, which are unable to perform nonribosomal peptide synthesis (NRPS) to generate enterobactin and its derivatives, are also sensitive to peroxide-mediated killing and cannot detoxify extracellular peroxide. In contrast to the dogma (26–28), siderophores were produced by *Salmonella* under iron-rich conditions when peroxide was included in the media. Our genetic dissection of the enterobactin processing pathways revealed that glucosylated products of enterobactin (salmochelin and its linearized forms) are not required for protection from peroxide, while esterases IroE and Fes are both required for this protection. Finally, we determined that the linearized enterobactin trimer (Ent-TRI) dihydroxybenzoylserine (DHBS)_3_ protects *Salmonella* from peroxide-mediated killing when added exogenously or from conditioned media of the wild-type organism containing peroxide. We conclude that (DHBS)_3_ is a natural substrate of MacAB and is a novel antioxidant responsible for enhancing *Salmonella* survival under inflammatory conditions during infection as well as during ROS exposure *in vitro*.

## RESULTS

### The MacAB efflux pump is required for survival in the inflamed intestines.

**FIG 1:**
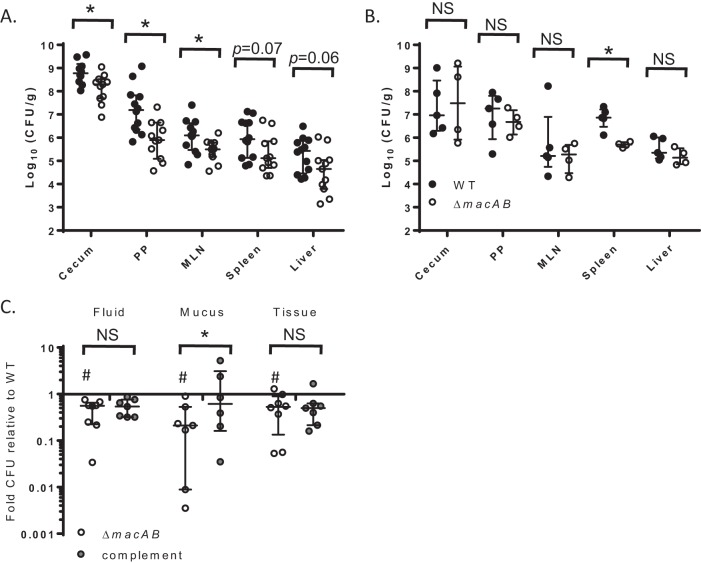
MacAB is required for survival in the inflamed intestines. (A and B) C57BL/6 mice were treated with streptomycin 24 h before infection (A) or left untreated (B). Animals in both groups were orally infected with approximately 10^8^ CFU of the wild-type strain (closed circles) or the Δ*macAB* mutant (open circles). Cecum, Peyer’s patches (PP), mesenteric lymph nodes (MLN), spleen, and liver were collected after 4 days of infection and analyzed for CFU enumeration. The asterisks indicate significance in Student’s *t* test with *P* values of <0.05. NS, not significant. (C) Calf ligated ileal loops were inoculated with approximately 10^7^ to 10^8^ CFU of the wild-type strain or the Δ*macAB* or Δ*macAB* pWSK29-*macAB* mutant strain. Fluid, mucus, and ileal tissue were collected from each loop after 12 h of infection and analyzed for CFU enumeration. Data are expressed as fold growth relative to the wild type. The crosshatch symbol (#) indicates significance in Student’s *t* test with *P* values of <0.05. The asterisk indicates statistical significance in 2-way analysis of variance (ANOVA) with Bonferroni’s multiple-comparison test.

Streptomycin treatment and subsequent *Salmonella* infection induce a strong neutrophilic influx into the intestinal tissue and lumen, mimicking the classical inflammatory diarrhea caused by NTS in humans (29). We have previously shown the importance of the MacAB efflux pump for *Salmonella* fitness in streptomycin-treated mice (15) (Fig. 1A), and the intestinal colonization defects of *macAB* mutants in this model are reversible by complementation in *trans* (Fig. S1). Furthermore, in C57Bl6 mice that were not pretreated with streptomycin, the MacAB efflux pump was dispensable for intestinal colonization (Fig. 1B). In untreated C57Bl6 mice, Δ*macAB* mutants poorly colonized the spleen, a niche where salmonellae reside within macrophages (Fig. 1B). These data support the hypothesis that MacAB is necessary for surviving in murine models that develop an intestinal inflammatory response or in cells where reactive oxygen species play a prominent role.

10.1128/mBio.00528-20.1 FIG S1 Complementation of *ΔmacAB* gene deletion with the wild-type copy of *macAB* genes on low-copy-number plasmid improves *Salmonella* survival in cecum, spleen, and liver. C57Bl/6 mice were treated with streptomycin 24 h before infection. Animals were orally infected with approximately 10^8^ CFU of the wild type (black circles), *ΔmacAB* mutant (open circles), or *ΔmacAB* pWSK29-*macAB* mutant (gray circles). Cecum, Peyer's patches (PP), mesenteric lymph nodes (MLN), spleen, and liver were collected after 4 days of infection and analyzed for CFU enumeration. The asterisk indicates significance in Student's *t* test with *P* values of <0.05. Download FIG S1, TIF file, 2.7 MB. Copyright © 2020 Bogomolnaya et al. 2020 Bogomolnaya et al. This content is distributed under the terms of the Creative Commons Attribution 4.0 International license.

In contrast to mice, calves are natural hosts for *Salmonella* that exhibit similar clinical and pathological signatures upon infection with *S*. Typhimurium, including neutrophilic infiltrates in the intestine, without any prior treatment (30). To further link the role of MacAB with survival of *Salmonella* during the inflammatory response in a host with an intact intestinal microbiota, we tested the fitness of a Δ*macAB* mutant in bovine ligated ileal loops. The Δ*macAB* mutant had a significantly reduced ability to replicate and/or survive in the luminal fluid, the mucus, and the intestinal tissue compared to the otherwise isogenic wild type (Fig. 1C). Complementation of the Δ*macAB* mutant with intact copies of the *macAB* genes restored the ability of the resulting strain to survive in the intestinal mucus (Fig. 1C). These data further support the hypothesis suggesting the necessity for MacAB in surviving oxidative stress during infection.

### Production of enterobactin but not salmochelin is required for protection against an oxidative stress.

MacA and MacB in salmonellae share 34% to 47% identity and 51% to 62% similarity to the corresponding proteins in Pseudomonas aeruginosa PAO-1, where they are linked to the export of products of nonribosomal peptide synthesis (NRPS) (31). On this basis, we hypothesized that the function of MacAB in *Salmonella* could be similar to its function in *Pseudomonas*, to export products of NRPS. In *Salmonella*, the siderophore enterobactin is synthesized via NRPS. Enterobactin is a cyclic trimer of 2,3-hydroxybenzyol serine, and the NRPS enzyme encoded by *entF* performs the last step in the synthesis of this molecule (32, 33). Enterobactin can be glucosylated into a second siderophore called salmochelin by IroB and/or further processed into several linear products (34, 35).

**FIG 2:**
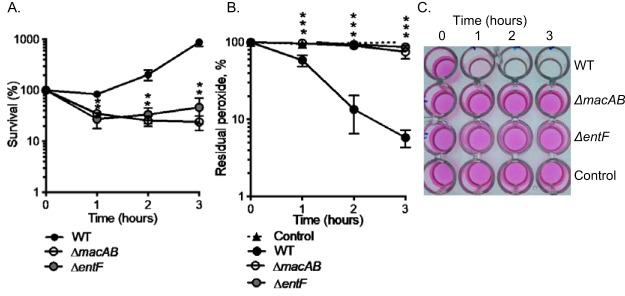
Production of siderophores is required for protection against an oxidative stress. (A) Overnight cultures of the wild-type strain (WT) (black circles), Δ*macAB* mutant (open circles), and Δ*entF* mutant (gray circles) were subcultured at 1/100 in LB containing 1 mM H_2_O_2_ and incubated at 37°C with aeration. Aliquots were collected hourly, serially diluted, and plated. The supernatants were retained for analysis in the experiments whose results are shown in panels B and C. Data are expressed as the survival means of results from at least three independent experiments with standard errors. (B and C) Supernatants collected from bacteria grown in peroxide-containing media as described in the panel A legend were tested for the residual H_2_O_2_ concentration using Amplex red reagent (Invitrogen). Sterile H_2_O_2_-containing medium (black triangle, dotted line) was included as a control. Data are expressed as means of levels of residual peroxide in the media from at least three independent experiments with standard errors (B). (C) Image representative of the results of the Amplex red assay. The concentration of peroxide is directly proportional to the pink color development as a result of Amplex red oxidation. The asterisks in panels A and B indicate significance in Student’s *t* test with *P* values of <0.05.

To determine whether the products of NRPS are linked to survival of *Salmonella* in the presence of reactive oxygen species (ROS), we evaluated the growth of an Δ*entF* mutant in medium containing hydrogen peroxide (1 mM). Mutants lacking EntF cannot produce enterobactin or any of its downstream products. Under these growth conditions, 70% of the bacteria in the Δ*entF* mutant culture were killed, a reduction in viability very similar to that seen with the Δ*macAB* mutant under similar conditions (Fig. 2A). In contrast, the wild-type organism resumed growth after 2 h of exposure to peroxide (Fig. 2A).

Because bacteria lacking the MacAB transporter are unable to decrease the concentration of H_2_O_2_ in the growth medium (15), we were interested in determining whether a mutant lacking *entF* would have a similar phenotype. Using the Amplex red assay, we determined that mutants lacking *entF* cannot detoxify extracellular peroxide (Fig. 2B and C). Collectively, these data indicate that *Salmonella* is sensitive to hydrogen peroxide in the absence of siderophore production and cannot reduce the amount of H_2_O_2_ in the growth media.

Finally, enterobactin can be glycosylated in *S.* Typhimurium by the glycosyltransferase IroB to form a second siderophore called salmochelin (34). In order to determine whether salmochelin plays a role in the protection of *Salmonella* from peroxide-mediated stress, we tested the ability of a mutant lacking *iroB* to resist peroxide-mediated killing and to detoxify extracellular peroxide. We found that the level of resistance of the salmochelin-deficient Δ*iroB* mutant to peroxide-mediated killing was similar to that seen with the wild-type strain grown under similar conditions (Table 1 and Fig. S2A). In the Amplex red assay, the Δ*iroB* mutant was able to efficiently reduce the concentration of peroxide in the growth medium to a level similar to that seen with wild-type strain (Table 1 and Fig. S3A). Collectively, our results support the hypothesis that it is enterobactin, or metabolites thereof, and not salmochelin or its metabolites which is involved in protection of *S*. Typhimurium against oxidative stress.

**TABLE 1:** Genetic analysis of siderophore-related mutants^*a*^

Strain	Biological function	H_2_O_2_ sensitivity^*c*^	Residual [H_2_O_2_]^*d*^
*S*. Typhimurium WT		167.7 ± 37.4	5.5 ± 1.7
*S*. Typhimurium Δ*macAB*	ABC-type efflux pump, substrate unknown	29.8 ± 6.3	81.6 ± 10.0
*S*. Typhimurium Δ*entF*	Required for synthesis of (E), (S), and all linear metabolites^*b*^	27.3 ± 9.4	83.6 ± 5.8
*S*. Typhimurium Δ*iroB*	Glucosyltransferase; converts (E) to (S)	83.3 ± 11.8	10.0 ± 8.1
*S*. Typhimurium Δ*entS*	ABC-type efflux pump for (E) export	230.6 ± 27.5	1.2 ± 0.2
*S*. Typhimurium Δ*iroC*	ABC-type efflux pump for (S) export	171.4 ± 65.6	1.5 ± 0.1
*S*. Typhimurium Δ*iroD*	Esterase; converts (E) and (S) to linear metabolites	128.2 ± 39.7	6.9 ± 2.9
*S*. Typhimurium Δ*iroE*	Esterase; converts (E) and (S) to linear metabolites	53.5 ± 13.6	28.9 ± 14.5
*S*. Typhimurium Δ*fes*	Esterase; converts (E) and (S) to linear metabolites	58.6 ± 3.5	55.5 ± 14.0
*S*. Typhimurium Δ*iroDE*		51.1 ± 9.0	23.4 ± 7.4
*S*. Typhimurium Δ*iroDE* Δ*fes*		21.0 ± 12.7	73.8 ± 16.9

a (E), enterobactin; (S), salmochelin; WT, wild type.

b Linear metabolites; see Fig. 6.

c After 2 h of growth in 1 mM H_2_O_2_, percent survival relative to *t*(0).

d After 2 h of growth in 1 mM H_2_O_2_, percent peroxide relative to *t*(0).

10.1128/mBio.00528-20.2 FIG S2 Esterases Fes and IroE but not IroD are involved in survival of *Salmonella* in peroxide-containing media. (A to E) Overnight cultures of the wild type (black circles) or *ΔmacAB* (open circles), *ΔiroB* (black squares), *ΔiroD* (black inverted triangle), *ΔiroE* (open inverted triangle), *ΔiroDE* (gray inverted triangle), *Δfes* (open triangle), and Δ*iroDE* Δ*fes* (gray triangle) mutants were subcultured 1/100 in LB containing 1 mM H_2_O_2_ and incubated at 37°C with aeration. Aliquots were collected hourly, serially diluted, and plated. The supernatants were retained for analysis in Fig. S3. Data are expressed as the survival means of at least three independent experiments with the standard error. The asterisk indicates significance in Student's *t* test with *P* values of <0.05. Download FIG S2, TIF file, 2.7 MB. Copyright © 2020 Bogomolnaya et al. 2020 Bogomolnaya et al. This content is distributed under the terms of the Creative Commons Attribution 4.0 International license.

10.1128/mBio.00528-20.3 FIG S3 Esterases Fes and IroE but not IroD are needed for extracellular peroxide detoxification. Supernatants collected from bacteria grown in peroxide-containing media (Fig. S2) were tested for the residual H_2_O_2_ concentration using Amplex Red reagent (Invitrogen). Sterile H_2_O_2_-containing media (black triangle, dotted line) was included as a control. Data expressed as a mean of residual peroxide in the media from at least three independent experiments with a standard error. The asterisk indicates significance in Student's *t* test with *P* values of <0.05. Download FIG S3, TIF file, 2.7 MB. Copyright © 2020 Bogomolnaya et al. 2020 Bogomolnaya et al. This content is distributed under the terms of the Creative Commons Attribution 4.0 International license.

### Secretion of the antioxidant molecule is independent of EntS and IroC efflux pumps.

**FIG 3:**
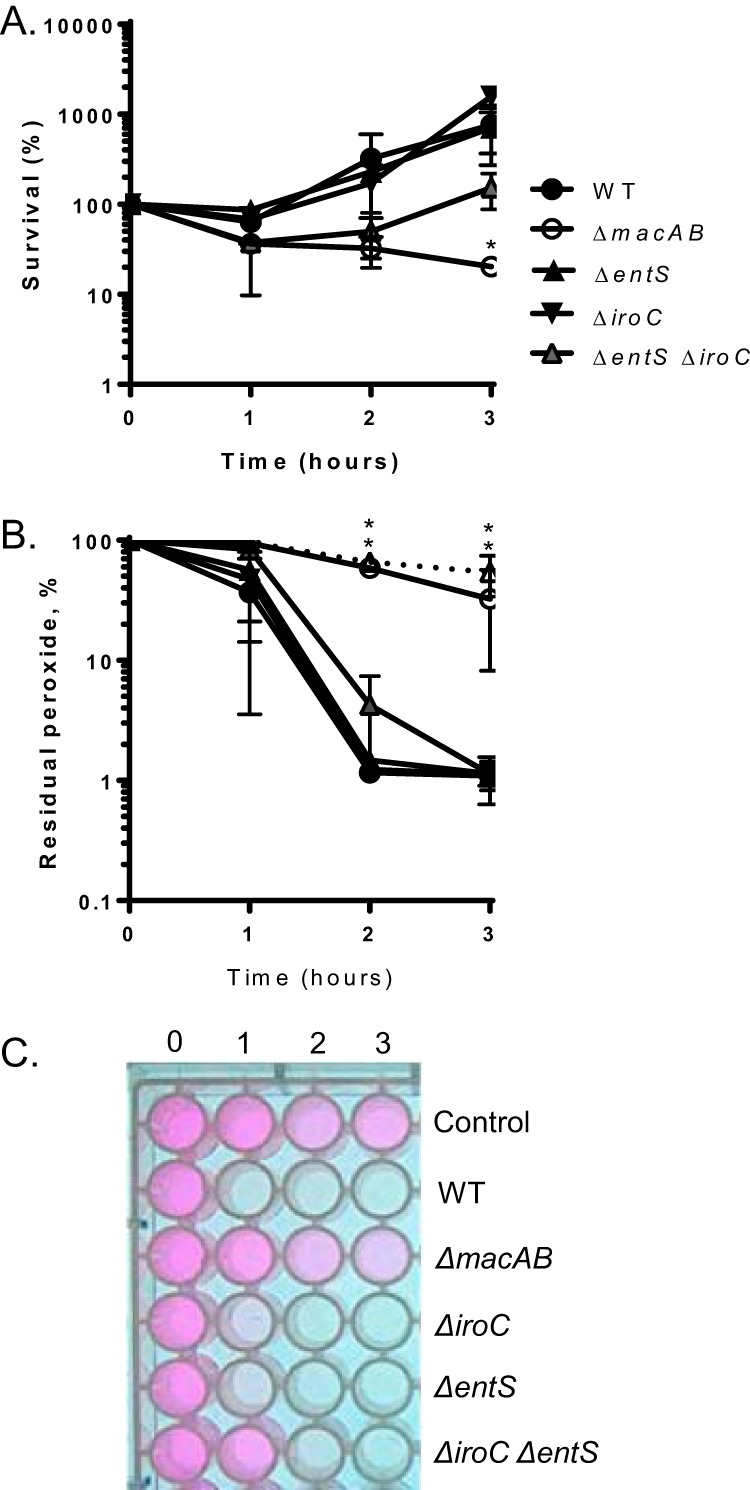
Secretion of antioxidant molecule is independent of EntS and IroC efflux pumps. (A) Overnight cultures of the wild-type strain (black circles) and the Δ*macAB* (open circles), Δ*entS* (black upward-pointing triangle), Δ*iroC* (black downward-pointing triangle), and Δ*entS* Δ*iroC* (gray upward-pointing triangle) mutants were subcultured at 1/100 in LB containing 1 mM H_2_O_2_ and incubated at 37°C with aeration. Aliquots were collected hourly, serially diluted, and plated. The supernatants were retained for analysis in the experiments whose results are presented in panels B and C. Data are expressed as the survival means of results from at least three independent experiments with standard errors. (B and C) Supernatants collected from bacteria grown in peroxide-containing media as described in the panel A legend were tested for the residual H_2_O_2_ concentration using Amplex red reagent (Invitrogen). Sterile H_2_O_2_-containing medium (black triangle, dotted line) was included as a control. Data are expressed as means of levels of residual peroxide in the media from at least three independent experiments with standard errors (B). Data corresponding to the *y* axis in panel B represent percent residual peroxide, and the data corresponding to the *x* axis represent time in hours. (C) Representative image of the Amplex red assay. The asterisks in panels A and B indicate significance in Student’s *t* test with *P* values of <0.05.

Secretion of enterobactin and salmochelin in *Salmonella* is mediated by two efflux pumps, EntS and IroC, respectively (36–38). We investigated whether (i) secretion of an antioxidant molecule during peroxide exposure relied on the presence of EntS and IroC and (ii) whether EntS and IroC efflux pumps act with MacAB in a common pathway. We found that deletion of Δ*entS* or Δ*iroC* did not change the hydrogen peroxide sensitivity of the mutant strains compared to that of the wild type (Fig. 3A). Double mutants lacking both efflux pumps were not statistically different in peroxide sensitivity relative to the wild-type organism (Fig. 3A). In the Amplex red assay, both the single mutants with a mutation in Δ*entS* or Δ*iroC* and the double mutant Δ*entS* Δ*iroC* reduced the extracellular peroxide concentration with kinetics similar to the results seen with the wild-type organism (Fig. 3B and C).

To validate our findings, we cocultured the Δ*macAB* mutant with the single or double mutations in siderophore efflux pumps in peroxide-containing media. We found that, similarly to the results seen with coculture experiments performed previously with wild-type *S.* Typhimurium (15), coculture with the Δ*entS* (Fig. S4A and B), Δ*iroC* (Fig. S4C and D), or Δ*entS*Δ*iroC* mutant (Fig. S4E and F) protected growth of the peroxide-sensitive Δ*macAB* mutant strain in the medium containing 1 mM H_2_O_2_ (data not shown). These results indicate that secretion of an antioxidant molecule was taking place in the absence of EntS and IroC efflux pumps. Thus, we conclude that the MacAB efflux pump functions independently of known siderophore efflux systems EntS and IroC.

10.1128/mBio.00528-20.4 FIG S4 MacAB functions independently of known siderophore pumps EntS and IroC. Overnight cultures of *ΔmacAB* (open circles), *ΔentS* (black triangle), Δ*iroC* (black inverted triangle), or Δ*entS* Δ*iroC* (gray triangle) mutants were subcultured at 1/100 in LB containing 1 mM H_2_O_2_ (A, C, E) or the individually grown Δ*macAB* mutant strain was first mixed with the equal amount of *ΔentS* (B), *ΔiroC* (D), or *ΔentS* *ΔiroC* (F), and the resulting mixtures were subcultured at 1/100 in peroxide-containing LB. Bacteria were incubated at 37°C with aeration. Aliquots were collected hourly, serially diluted, and plated for CFU enumeration. Data are presented as mean survival from at least three independent experiments. The asterisk indicates significance in the Student's *t* test with *P* values of <0.05. Download FIG S4, TIF file, 2.7 MB. Copyright © 2020 Bogomolnaya et al. 2020 Bogomolnaya et al. This content is distributed under the terms of the Creative Commons Attribution 4.0 International license.

### For growth in and detoxification of extracellular peroxide, *entF* and *macAB* function in the same genetic pathway.

We investigated whether *entF* and *macAB* function in the same pathway or in parallel pathways to mitigate the effects of peroxide-mediated stress. Coculture with wild-type *Salmonella* protects Δ*macAB* mutants from peroxide-mediated killing (15). In order to determine whether an *entF* mutant could be similarly protected by the presence of wild-type bacteria, we mixed individually grown overnight cultures of the wild type and the Δ*entF* mutant in equal ratios and subcultured the mixture into fresh medium containing 1 mM H_2_O_2_.

**FIG 4:**
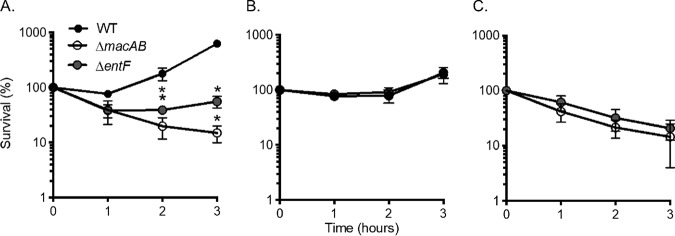
*entF* and *macAB* function in the same genetic pathway. (A) Overnight cultures of the wild-type strain (black circles) and the Δ*macAB* (open circles) and Δ*entF* (gray circles) mutants were subcultured at 1/100 in LB containing 1 mM H_2_O_2_ and incubated at 37°C with aeration. Aliquots were collected hourly, serially diluted, and plated for CFU enumeration. Data are presented as mean survival results from at least three independent experiments. (B and C) Individually grown overnight cultures of the wild-type strain and the Δ*entF* mutant (B) or Δ*macAB* and Δ*entF* mutants (C) were mixed in equal ratios, and the resulting mixtures were subcultured in peroxide-containing LB and grown at 37°C with aeration. Aliquots were processed as described for panel A. The asterisk indicates significance in Student’s *t* test with *P* values of <0.05.

In contrast to individual cultures grown in the presence of peroxide (Fig. 4A), the presence of the wild-type organism protected the Δ*entF* mutant from peroxide-mediated killing (Fig. 4B). To rule out the possibility that *entF* and *macAB* function in parallel pathways, we mixed individually grown overnight cultures of Δ*macAB* and Δ*entF* mutant strains and tested the survival of both organisms in peroxide as described above. Coculture of the siderophore-deficient mutant with the Δ*macAB* mutant failed to protect either strain from peroxide-mediated killing (Fig. 4C). Taken together, these experimental results indicate that EntF and MacAB function in a common pathway to mitigate the effects of peroxide-mediated stress.

### Exogenous enterobactin cannot rescue Δ*macAB* mutant cells from peroxide-mediated death.

**FIG 5:**
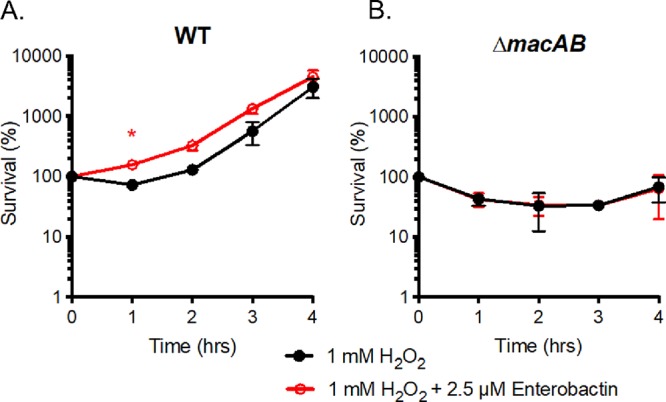
Enterobactin cannot rescue the Δ*macAB* mutant from peroxide-mediated death. (A and B) Overnight cultures of the wild-type strain (A) or the Δ*macAB* mutant (B) were subcultured at 1/100 in peroxide-containing LB or in 1 mM H_2_O_2_ with LB additionally supplemented with 2.5 μM enterobactin and incubated at 37°C with aeration. Aliquots were collected hourly, serially diluted, and plated for CFU enumeration. Data are presented as mean survival results from at least three independent experiments. The asterisk indicates significance in Student’s *t* test with *P* values of <0.05.

EntF is essential for the production of enterobactin, and, based on the results described above, the simplest hypothesis is that enterobactin itself might be responsible for mitigating peroxide-mediated effects on *S*. Typhimurium. In order to test this idea, we supplemented peroxide-containing growth medium with exogenous enterobactin and used this medium to test for survival and growth of various isolates. Supplementation of peroxide-containing medium with exogenous enterobactin promoted growth of wild-type *S*. Typhimurium (Fig. 5A). However, exogenous enterobactin added in a range of concentrations from 2.5 to 25 μM failed to rescue the growth of the Δ*macAB* mutant in the presence of 1 mM H_2_O_2_ (Fig. 5B and data not shown). This result suggested that enterobactin itself is not protective during peroxide-mediated stress and is not the substrate of the MacAB efflux pump.

### Esterases Fes and IroE, but not IroD, are involved in protection of *S*. Typhimurium against hydrogen peroxide.

**FIG 6:**
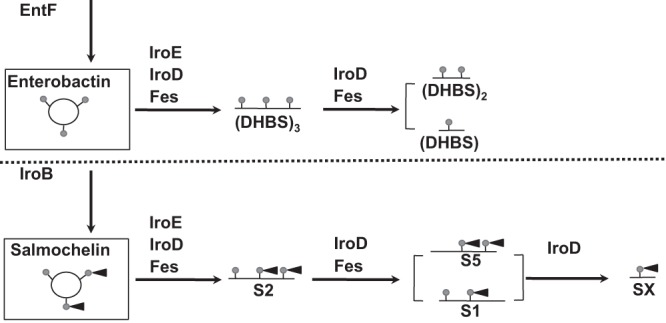
Schematic overview of the siderophore pathway. DHBS, 2,3-dihydroxybenzoylserine, enterobactin monomer (Ent-Mono); (DHBS)_2_, enterobactin dimer (Ent-Dim); (DHBS)_3_, linear enterobactin trimer (Ent-TRI); SX, glucosylated 2,3-dihydroxybenzoylserine (pacifarin acid); S1 and S5, glucosylated enterobactin dimers; S2, glucosylated linear enterobactin trimer.

Enterobactin can be further hydrolyzed to linear metabolites by the esterases Fes, IroE, and IroD (Fig. 6). To determine which of these enzymes and, potentially, which linear metabolites play a role in protection of salmonellae against an oxidative stress, we generated deletion mutants in *fes*, *iroE*, and *iroD*. Each mutant strain was tested for two phenotypes associated with the loss of MacAB efflux pump: (i) sensitivity to hydrogen peroxide and (ii) the ability to degrade extracellular H_2_O_2_ (15). First, as expected, the loss of the esterase IroD, which preferably hydrolyses iron-loaded salmochelin (39), did not affect *S.* Typhimurium sensitivity to peroxide or the ability to reduce concentration of H_2_O_2_ in the medium (Table 1 and Fig. S2B and S3B). In contrast, deletion of *iroE* both moderately increased sensitivity to peroxide and slowed the degradation of H_2_O_2_ in the medium in Amplex red assay (Table 1 and Fig. S2C and S3C). Similarly to the loss of IroE, the loss of Fes resulted in a moderate increase in peroxide sensitivity and led to a substantial delay in H_2_O_2_ degradation in Amplex red assay (Table 1 and Fig. S2D and S3D). A double mutant lacking the ability to produce both IroE and Fes recapitulated the phenotype of a *macAB* mutant for both peroxide sensitivity and peroxide degradation (Table 1 and Fig. S2E and S3E). Taken together, these experiments indicate that esterases IroE and Fes, but not IroD, are involved the production of an enterobactin metabolite that has a function in protection of *S*. Typhimurium against hydrogen peroxide.

### Siderophores and their derivatives are produced in iron-rich medium, and enterobactin trimer (Ent-TRI) protects *S*. Typhimurium against oxidative stress.

Siderophores are secreted by bacteria under iron-restricted conditions to acquire Fe(III) from the environment. Siderophores are also involved in protection of bacteria against oxidative stress (23) (Fig. 2). Luria-Bertani (LB) broth contains approximately 5.32 to 7.55 μM iron (40, 41), an amount sufficient to maintain growth of siderophore-deficient mutants (42). To show that siderophores are indeed secreted under iron-rich conditions and under conditions of oxidative stress, we grew wild-type *Salmonella* or the Δ*macAB* mutant strain in LB broth containing 0.5 mM H_2_O_2_ and isolated siderophores from the resulting conditioned media, which had been filtered to remove bacteria. The concentration of catecholate siderophores in the conditioned media was determined by Arnow assay and adjusted to 2.5 μM (43).

**FIG 7:**
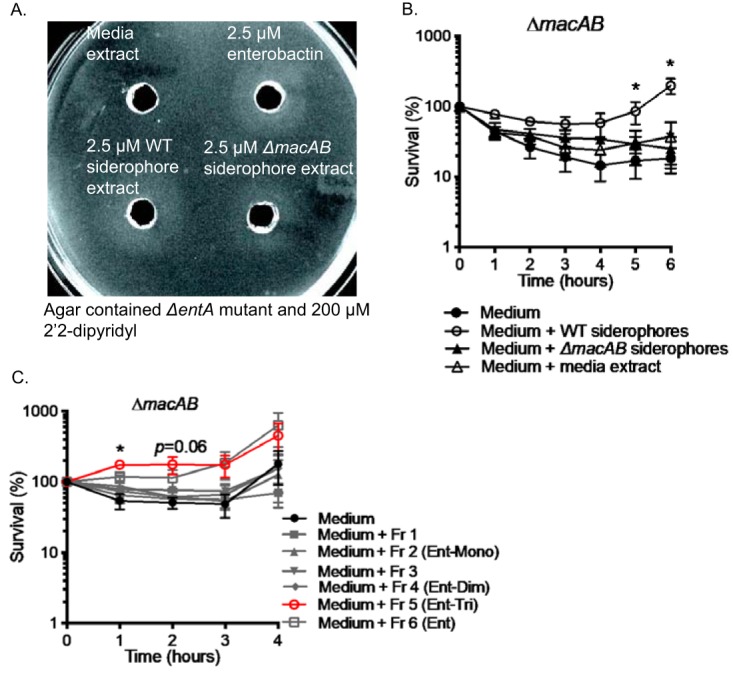
Siderophores and their derivatives are produced in iron-rich media, and Ent-TRI protects *Salmonella* against oxidative stress. (A) Siderophores extracted from the conditioned broth with the wild-type strain and the Δ*macAB* mutant exposed to peroxide were tested in cross-feeding assays for their ability to support growth of siderophore-deficient Δ*entA* mutant cells on the plate containing iron chelator 2′2-dipyridyl (200 μM). Purified enterobactin (2.5 μM) and mock siderophore extract were used as the positive control and negative control, respectively. A halo around a well indicates the presence of siderophore in the test sample. The picture was taken after 24 h of incubation at 37°C. (B) An overnight culture of the Δ*macAB* mutant was subcultured at 1/100 in peroxide-containing LB (black circles) or in the same media additionally supplemented with siderophore extract from the media containing the wild type (open circles), from Δ*macAB-*conditioned media (black triangles), and from mock extract (open triangles) and incubated at 37°C with aeration. Aliquots were collected hourly, serially diluted, and plated for CFU enumeration. (C) An overnight culture of Δ*macAB* mutant was subcultured at 1/100 in peroxide-containing LB (black circles) or in the same media additionally supplemented with HPLC-separated fractions (Fr) of siderophore extract from wild-type-conditioned media (as described in Materials and Methods). The resulting cultures were incubated at 37°C with aeration. Aliquots were processed as described for panel B. Data in panels B and C are presented as mean survival results from at least three independent experiments. The asterisk indicates the significance in Student’s *t* test with *P* values of <0.05.

Cross-feeding assays were used to determine whether extracts of the conditioned medium described above contained siderophores in concentrations sufficient to rescue the growth of siderophore-deficient Δ*entA* mutants. Siderophore extracts prepared from both the wild-type-conditioned media and the Δ*macAB* mutant-conditioned media, but not a “mock” extract from uninoculated LB broth, supported the growth of siderophore-deficient Δ*entA* mutants on agarized medium containing the iron chelator 2′2-dipyridyl (200 μM) (Fig. 7A). This result suggests that siderophores are secreted by *Salmonella* when peroxide is present in the medium, even under iron-rich conditions.

Next, we investigated whether siderophores in these extracts would be able protect an *S.* Typhimurium Δ*macAB* mutant against hydrogen peroxide-mediated death. First, we confirmed that the extracts were not toxic to *Salmonella* (Fig. S5). Despite the presence of siderophores in extracts prepared from both wild-type-conditioned and Δ*macAB* mutant-conditioned media (Fig. 7A), only the extract from the wild type had a protective effect on the growth of the Δ*macAB* mutant in peroxide-containing media (Fig. 7B). This finding suggests that enterobactin and salmochelin, secreted via the ABC-type transporters IroC and EntS, are not themselves the critical molecules for protection that are being secreted by *Salmonella* against oxidative stress. We hypothesized that derivatives of enterobactin could be playing a role in the protection of *Salmonella* against oxidative stress.

10.1128/mBio.00528-20.5 FIG S5 Siderophore extracts are not toxic to wild-type *Salmonella* exposed to peroxide. Overnight culture of the wild type was subcultured at 1/100 in peroxide-containing LB (black circles) or in the same media additionally supplemented with siderophore extract from wild-type (open circles), *ΔmacAB*-conditioned media (black triangles), and mock extract (open triangles) and incubated at 37°C with aeration. Aliquots were collected hourly, serially diluted, and plated for CFU enumeration. Data are presented as mean survival from at least three independent experiments. Download FIG S5, TIF file, 2.7 MB. Copyright © 2020 Bogomolnaya et al. 2020 Bogomolnaya et al. This content is distributed under the terms of the Creative Commons Attribution 4.0 International license.

To identify the enterobactin derivatives responsible for protection against reactive oxygen species, we separated siderophore extracts by high-pressure liquid chromatography (HPLC) and collected fractions expected to contain the following enterobactin derivatives: enterobactin monomer (Ent-Mono; 2,3-dihydroxybenzoylserine [DHBS]) (fraction 2); enterobactin dimer [Ent-Dim; (DHBS)_2_] (fraction 4); enterobactin trimer [Ent-TRI; (DHBS)_3_] (fraction 5), and enterobactin cyclic trimer (fraction 6). The enterobactin derivatives expected in each fraction were determined in advance by running purified commercially available derivatives of enterobactin on HPLC to determine their retention times. For negative controls, we collected two fractions (fractions 1 and 3) that were not expected to contain any siderophores. Each fraction was tested for its ability to rescue the hydrogen peroxide sensitivity of the Δ*macAB* mutant. Only fraction 5 prepared from the wild-type siderophore extract, expected to contain Ent-TRI, had a statistically significant protective effect on the survival of the Δ*macAB* mutant grown in the presence of peroxide (Fig. 7C). In contrast, the corresponding fraction prepared from Δ*macAB* siderophore extract failed to support growth of the Δ*macAB* mutant in peroxide-containing medium (data not shown).

### Exogenous linearized enterobactin protects the *S*. Typhimurium Δ*macAB* mutant from hydrogen peroxide-mediated killing.

**FIG 8:**
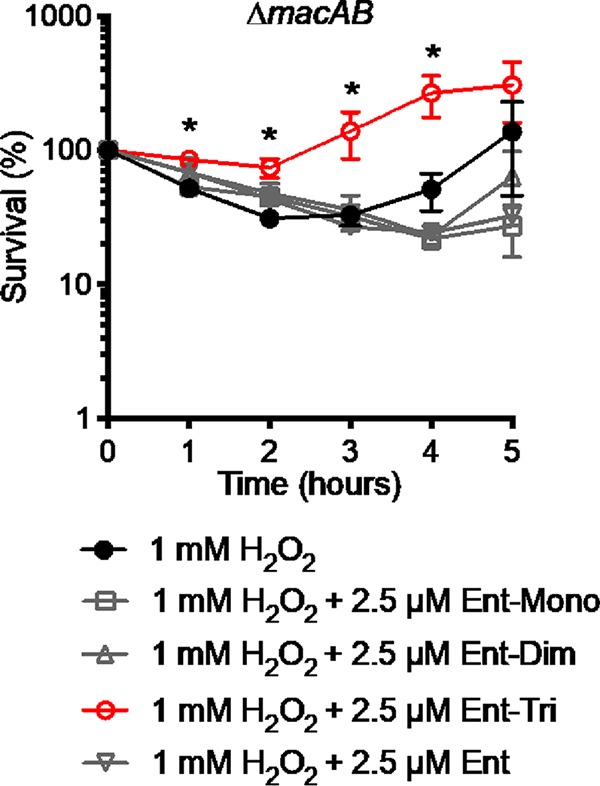
Exogenous linearized enterobactin protects Δ*macAB* mutant cells from hydrogen peroxide-mediated killing. An overnight culture of the Δ*macAB* mutant was subcultured at 1/100 in peroxide-containing LB (black circles) or in the same media additionally supplemented with enterobactin monomer (gray open squares), enterobactin dimer (gray open upward-pointing triangles), enterobactin trimer (open circles), and enterobactin (gray open downward-pointing triangles) and incubated at 37°C with aeration. Aliquots were collected hourly, serially diluted, and plated for CFU enumeration. Data are presented as mean survival results from at least three independent experiments. Asterisks indicate significance in Student’s *t* test with *P* values of <0.05.

To confirm whether purified enterobactin derivatives could protect a *macAB* mutant from peroxide-mediated killing, we used commercially available purified enterobactin derivatives, i.e., Ent-Mono, Ent-Dim, Ent-TRI (EMC Microcollections), and Ent (Sigma), in a peroxide survival assay. Each siderophore derivative was used at 2.5 μM. In agreement with our previous observations (Fig. 7C), neither Ent-Mono nor Ent-Dim was able to rescue growth of the peroxide sensitive Δ*macAB* mutant in medium containing 1 mM H_2_O_2_. In contrast, supplementation of peroxide-containing medium with 2.5 μM Ent-TRI significantly improved the survival of the Δ*macAB* mutant (Fig. 8). The results of this experiment suggest that linearized enterobactin trimer is the substrate of MacAB efflux pump and that this molecule can protect *Salmonella* from damaging reactive oxygen species.

### Linearized enterobactin protects *S*. Typhimurium Δ*iroE* Δ*fes* esterase mutants from hydrogen peroxide.

Given that active esterases IroE and Fes are needed for protection of *S*. Typhimurium against peroxide-mediated damage (Table 1 and Fig. S2E and S3E), we investigated whether they work in the same pathway with the MacAB efflux pump. As expected, the Δ*iroE* Δ*fes* mutant was more sensitive to peroxide-mediated killing than the wild type (Fig. S6A). Coculture of the Δ*iroE* Δ*fes* mutant with the wild type in the presence of H_2_O_2_ resulted in improved survival of the mutant (Fig. S6B). In contrast, coculture of the Δ*iroE* Δ*fes* and the Δ*macAB* mutants in the presence of peroxide did not improve the survival of esterase mutant (Fig. S6C). These results indicate that IroE and Fes function in the same pathway as MacAB.

10.1128/mBio.00528-20.6 FIG S6 *iroE* and *fes* function in the same genetic pathway with *macAB*. (A) Overnight cultures of the wild type (black circles) and *ΔmacAB* (black triangle) and *ΔiroE* *Δfes* (open circles) mutants were subcultured at 1/100 in LB containing 1 mM H_2_O_2_ and incubated at 37°C with aeration. Aliquots were collected hourly, serially diluted, and plated for CFU enumeration. Data are presented as mean survival from at least three independent experiments. (B and C) Individually grown overnight cultures of the wild type and *ΔiroE* *Δfes* mutant (B) or *ΔmacAB* and *ΔiroE* *Δfes* mutants (C) were mixed in equal ratios, and the resulting mixtures were subcultured in peroxide-containing LB and grown at 37°C with aeration. Aliquots were processed as described for panel A. The asterisk indicates significance in Student's *t* test with *P* values of <0.05. Download FIG S6, TIF file, 2.7 MB. Copyright © 2020 Bogomolnaya et al. 2020 Bogomolnaya et al. This content is distributed under the terms of the Creative Commons Attribution 4.0 International license.

**FIG 9:**
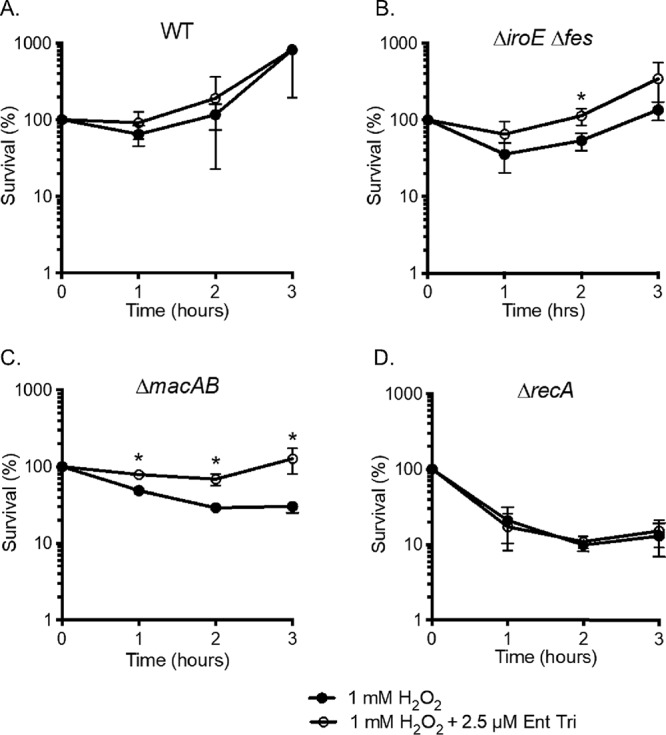
Linearized enterobactin protects Δ*iroE* Δ*fes* mutant cells from hydrogen peroxide-mediated killing. Overnight cultures of the wild-type strain (A) and the Δ*iroE* Δ*fes* (B), Δ*macAB* (C), and Δ*recA* (D) mutants were subcultured at 1/100 in peroxide-containing LB (black circles) or in the same media additionally supplemented with enterobactin trimer (open circles) and incubated at 37°C with aeration. Aliquots were collected hourly, serially diluted, and plated for CFU enumeration. Data are presented as mean survival results from at least three independent experiments. Asterisks indicate significance in Student’s *t* test with *P* values of <0.05.

We hypothesize that the linearized enterobactin, Ent-TRI, a compound that protects the Δ*macAB* mutant from peroxide-mediated killing (Fig. 8), would also protect the Δ*iroE* Δ*fes* esterase mutant. Similarly to the Δ*macAB* mutant (Fig. 9C), supplementation of peroxide-containing LB broth with 2.5 μM Ent-TRI significantly improved the survival of the Δ*iroE* Δ*fes* double mutant (Fig. 9B). To ensure that Ent-TRI selectively protects mutants with mutations in genes that belong to MacAB pathway, we tested the effect of linearized enterobactin on the unrelated peroxide-sensitive Δ*recA* mutant. Mutation in *recA* abrogates the ability of *Salmonella* to repair peroxide-damaged DNA, and this eventually results in bacterial death. Supplementation of peroxide-containing media with Ent-TRI did not improve the survival of the Δ*recA* mutant (Fig. 9D). Taken together, these experiments demonstrated that linearized enterobactin trimer, secreted by *Salmonella* under iron-rich conditions when peroxide is present, protects *S.* Typhimurium against peroxide-mediated killing in a MacAB-dependent fashion.

## DISCUSSION

Multidrug efflux pumps play an important role in survival of bacteria during antibiotic therapy (44). These pumps are usually chromosomally encoded membrane protein complexes that belong to the ancient families of proteins (45). Multiple efflux pumps with overlapping specificities to drugs are usually present in the single bacterial genome, which suggests that these protein complexes may have additional natural functions unrelated to the role in efflux of antimicrobials.

We showed previously that MacAB, an ABC-type efflux pump, originally linked to macrolide resistance and later to virulence in mice (9, 14), is involved in protection of *Salmonella* Typhimurium against oxidative stress (15). Reactive oxygen species are generated by the mammalian host in response to bacterial infection (46). To model human enteritis in response to *Salmonella* infection, we utilized two animal models: streptomycin-treated mice that develop colitis in response to infection and ligated ileal loops in calves, a model that closely mimics the clinical and pathological changes occurring during the early phase of salmonellosis. In agreement with earlier work (15), the mutant lacking MacAB was attenuated in both models in the inflamed intestines, indicating that this pump facilitates survival of *Salmonella* during infection.

The MacAB efflux pump was previously linked to secretion of heat-stable enterotoxin II produced by enterotoxigenic E. coli (12) and was suggested to be involved in export of the heme precursor protoporphyrin PPIX in E. coli when bacterial heme homeostasis is disrupted by iron starvation (13). MacAB homologs in *Pseudomonas* species were previously shown to be involved in secretion of lipopeptides (arthrofactin, putisolvins I and II, xantholysin, syringomycin, and syringopeptin) (47–50) and the siderophore pyoverdin (31). Both lipopeptide and siderophore molecules are synthesized nonribosomally through the function of multimodular nonribosomal peptide synthetases (NRPS). In the genomes of these bacteria, *macAB* genes are located downstream of the NRPS cluster. The *Salmonella* genome encodes a single NRP synthetase (EntF) that is necessary for siderophore synthesis (51). The genes that encode MacAB efflux pump, however, are located in an independent region of the genome. Nevertheless, the loss of EntF led to a drastic increase in hydrogen peroxide sensitivity and, similarly to the loss of MacAB efflux pump (15), to an inability to degrade extracellular H_2_O_2_. Under iron-limiting conditions, *Salmonella* produces two siderophores, enterobactin and its glycosylated derivative, salmochelin (35).

Catecholate siderophores were recently proposed to have an antioxidant role in addition to their well-established role in iron chelation (23–25), and it was also proposed that there is a link between the components of oxidative stress response and siderophore biosynthesis (52). In agreement with previous observations (23), the production of salmochelin was dispensable for *Salmonella* survival in peroxide. Surprisingly, the addition of exogenous enterobactin did not protect Δ*macAB* mutants against peroxide-mediated killing. This finding allowed us to conclude that enterobactin is not the natural substrate of MacAB, supported by the fact that the pathway for enterobactin export via EntS is well established (37).

Enterobactin can be further processed by esterases IroD, IroE, and Fes to produce shorter linear products (39). Even though these proteins are homologous to each other, they differ in their substrate specificities and in their cellular locations (39, 53). IroD is located in the cytoplasm and cleaves iron-loaded salmochelin (39). We found that IroB, the enzyme that glucosylates enterobactin to form salmochelin (54), was not required for protection of *Salmonella* against peroxide. We also found that the dedicated transporter for salmochelin, IroC (36), is not required for protection from ROS. Finally, the loss of IroD did not affect *S.* Typhimurium survival in the presence of peroxide. Thus, neither salmochelin nor its degredation products (S2, S1, and SX) could be a substrate of MacAB.

Fes and IroE, located in the cytoplasm and periplasm, respectively, hydrolyze both enterobactin and salmochelin (39). In addition, IroE hydrolyses iron-free siderophores to produce linearized trimer (DHBS)_3_, while Fes prefers iron-loaded siderophores to sequentially produce linearized trimer, dimer, and monomer degradation products. We found that both IroE and Fes are necessary to provide protection against hydrogen peroxide similar to the protection provided by organisms with intact MacAB and EntF systems. Consistent with these findings, *entE* and *fes* are also required for protection from oxidative killing in E. coli, although E. coli does not encode a homolog of IroE (24). Thus, both synthesis of enterobactin and its processing to a linear product are required for protection against ROS. Finally, a considerable amount of the linear products produced by the action of these esterases had long been known to be secreted from the bacterial cell, and yet the machinery required for their export remained unknown (36).

One cannot abrogate the production of endogenously produced enterobactin in *S.* Typhimurium to define the importance of this metabolite during infection, because it is a precursor to salmochelin and other linear products. However, several lines of evidence suggest that the primary purpose of enterobactin production by *Salmonella* during infection may not be its siderophore activity. First, enterobactin is produced by many *Enterobacteriaceae* (55) and is present in the murine intestine, where iron is not limited (55, 56). Second, deletion of *entS*, a gene encoding a key protein involved in the export of enterobactin, does not significantly reduce *Salmonella* virulence in murine models (36). Third, lipocalin-2, a host molecule secreted by several cell types, including neutrophils, binds iron-loaded enterobactin to sequester it from bacteria but is unable to bind salmochelin (57). Finally, while IroN and FepA, receptors on the cell surface that bind enterobactin prior to import, reduce *Salmonella* virulence when deleted (58), these receptors may also be involved in siderophore piracy of enterobactin produced by other enterobacteriaceae in the intestine and other siderophores, including corynebactin and myxochelin (59). Our data suggest that enterobactin serves not only as a precursor for salmochelin but also as a precursor for the production of linear products of enterobactin that are secreted via MacAB and whose main purpose may be neutralizing reactive oxygen species outside the bacterial cell during infection.

While the precise mechanism of this anti-H_2_O_2_ protection is not yet defined, there are at least two possible scenarios: linearized enterobactin trimer can either exhibit catalase-like activity or participate in ROS scavenging. An unrelated siderophore, yersiniabactin, complexed with copper, Cu(II)-Ybt, was recently shown to have superoxide dismutase (SOD)-like activity, supporting survival of uropathogenic E. coli within macrophages (60). Another example of SOD mimic compounds includes metalloporphyrins and salens that also possess catalase-like activity (61–63). Among all the compounds mentioned above, catalytic activity depends on the presence of redox-active metal: copper, manganese, or iron. The requirement for iron in enterobactin trimer-mediated peroxide detoxification is currently unknown. The second potential mechanism for Ent-TRI detoxification of reactive oxygen species is based on the well-known antioxidant activity of catechol moieties (64–68). These moieties are present and exposed that in the linearized enterobactin trimer. Elucidation of the detailed mechanism of the antiperoxide action of this molecule will be a fascinating area of future work.

## MATERIALS AND METHODS

### Bacterial strains and media.

All of the Salmonella enterica serovar Typhimurium strains used for this study are listed in Table 2 and were derived from ATCC 14028. Mutants were generated by lambda-Red recombinase-mediated homologous recombination (69, 70). HA420 is a fully virulent, spontaneously nalidixic acid-resistant (Nal^r^) derivative of ATCC 14028 (71). Deletion mutants used in this study were moved into a clean genetic background using P22 transduction (72).

**TABLE 2:** Strain and plasmid list

Plasmid	Strain^*a*^	Reference or source
HA420	ATCC 14028, spontaneously Nal^r^	71
HA995	ATCC 14028 Δ*macAB*::Cm^r^	14
LB321	ATCC 14028 Δ*macAB*::Cm^r^ pWSK29-*macAB*, Amp^r^	This study
LB329	HA420 Δ*entF*::Kan^r^	This study
JE384	HA420 Δ*iroB*::Kan^r^	This study
JE374	HA420 Δ*entS*::Kan^r^	This study
JE418	HA420 Δ*iroC*::Cm^r^	This study
JE419	HA420 Δ*entS*::Kan^r^ Δ*iroC*::Cm^r^	This study
JE430	HA420 Δ*iroD*::Kan^r^	This study
JE424	HA420 Δ*fes*::Cm^r^	This study
LB437	HA420 Δ*iroE*::Cm^r^	This study
LB444	HA420 Δ*iroDE*::Kan^r^	This study
LB649	HA420 Δ*iroE*::Kan^r^ Δ*fes*::Cm^r^	This study
LB500	HA420 Δ*iroDE*::Kan^r^ Δ*fes*::Cm^r^	This study
LB786	ATCC 14028 Δ*entA*::Kan^r^	This study
LB250	HA420 Δ*recA-hydN*::Kan^r^	15

a Amp^r^, ampicillin resistance; Cm^r^, chloramphenicol resistance; Kan^r^, kanamycin resistance; Nal^r^, nalidixic acid resistance.

Strains were routinely cultured in Luria-Bertani (LB) broth and plates, supplemented with antibiotics as needed at the following concentrations: 50 mg/liter nalidixic acid, 100 mg/liter carbenicillin, 50 mg/liter kanamycin, or 20 mg/liter chloramphenicol. For calf ligated ileal loop infections, strains were grown overnight at 37°C with aeration (200 rpm) in LB broth with appropriate antibiotic, subcultured 1/100 in fresh media, and grown for additional 3 h at 37°C. Bacteria were pelleted by centrifugation and washed twice in sterile LB, and the bacterial concentration was adjusted to approximately 10^7^ to 10^8^ CFU in 3 ml LB. For infection of *Salmonella*-susceptible C57BL/6 mice, strains were grown aerobically at 37°C to stationary phase in LB broth with an appropriate antibiotic.

### Plasmid construction.

A complementing plasmid carrying intact *macAB* genes was generated as follows. A DNA fragment containing the full-length open reading frames with approximately 300 bp upstream and downstream of *macAB* was amplified by PCR using macAB-EcoRI-Fwd (5′-GCGAATTCAGTAACGTATTTAACTCC-3′) as a forward primer and macAB-KpnI-Rev (5′-ATGGTACCAAGTGGTTCAACAATGCC-3′) as a reverse primer. PCR products were digested with EcoRI and KpnI (New England Biolabs) and ligated into pWSK29 vector (73) that had been previously digested with the same enzymes. Clones with the correct insertion were confirmed by restriction digestion and sequencing.

### Calf ligated ileal loop infection model.

All experiments involving animals described in this work were carried out in accordance with the recommendations in the Guide for the Care and Use of Laboratory Animals of the National Institutes of Health and were approved by the Institutional Animal Care and Use Committee at Texas A&M University or at North Carolina State University. *Salmonella* infections were performed as previously described (74). Briefly, Angus cross calves were separated from the dam at 1 day of age and fed milk replacement twice a day and were provided with access to water and grass hay. Fecal samples were collected weekly and were tested for the presence of *Salmonella* as described previously (30, 75). When they reached 3 to 6 weeks of age, the calves were anesthetized for ligated ileal loop surgery and ileal loops were tied and injected with 3 ml of approximately 10^7^ to 10^8^ CFU of wild-type or Δ*macAB* mutant *Salmonella* Typhimurium or with 3 ml of sterile LB broth. Ligated loops were returned to the abdominal cavity, incisions were closed, and calves were kept under anesthesia for the duration of the experiment. At the end of incubation period, individual loops were excised. Calves were humanely euthanized after all loops were excised as described previously (74). Intestinal fluid, mucus, and tissue samples were collected from each loop. Fluid volume, which correlates with the level of inflammation in the intestines (76), was determined by weighing each loop before and after fluid collection. Mucus was scraped from the luminal surface of the intestine and placed in 3 ml of sterile phosphate-buffered saline (PBS). The remaining tissue was placed in 5 ml PBS. All specimens were homogenized (IKA T25 Basic S1), serially diluted in PBS, and plated for CFU enumeration. To compare the bacterial burden in the loops infected with wild-type *Salmonella* to the CFU from the loops infected with mutant strain, data were expressed as fold growth as follows: [CFU(*t*_12_)/CFU(*t*_0_)]_mutant_/[CFU(*t*_12_)/CFU(*t*_0_)]_wild type_. Statistical significance was determined using Student’s two-tailed *t* test with significance set at a *P* value of <0.05.

### Oral infection of *Salmonella*-susceptible mice.

A *macAB* null mutant and a virulent HA420 strain (spontaneously Nal^r^) were tested for their ability to colonize 8-to-10-week-old female C57BL/6 mice (Jackson Laboratory) using the following protocol. Overnight cultures to be used as inocula were grown at 37°C with aeration, serially diluted, and plated for CFU to determine the exact titer.

Groups of five mice were infected by gavage with approximately 1 × 10^8^ bacteria of either the wild-type strain or the Δ*macAB* mutant strain in 100 μl of LB broth. At 4 days postinfection, mice were humanely euthanized and livers, spleens, Peyer’s patches, mesenteric lymph nodes, and ceca of infected mice were excised and homogenized in 3 ml PBS. Organ homogenates were weighed, serially diluted, and plated to determine bacterial burden in the infected tissues. Data are expressed as CFU counts per gram of tissue converted to a logarithmic scale. Statistical significance was determined using a Student’s *t* test and *P* values set to less than 0.05.

### Oral infections in the murine colitis model.

Female C57BL/6 mice (Jackson Laboratories) (8 to 10 weeks of age) were treated with 20 mg of streptomycin by gavage 24 h prior to infection. The wild-type strain (HA420), the Δ*macAB* mutant, and the Δ*macAB* mutant complemented with an intact copy of *macAB* were grown to stationary phase at 37°C with aeration. Cultures were serially diluted and plated to determine the exact titer of each strain used to inoculate animals.

Groups of five mice were inoculated by gavage with approximately 1 × 10^8^ bacteria in 100 μl. At 4 days postinfection, mice were humanely euthanized and livers, spleens, Peyer’s patches, mesenteric lymph nodes, and ceca of infected mice were excised and homogenized in 3 ml of PBS and serially diluted, and bacteria were enumerated. Data are expressed as CFU counts per gram of tissue, converted logarithmically. Statistical significance was determined using a Student’s *t* test and *P* values set less to than 0.05.

### Sensitivity of individual isolates to hydrogen peroxide.

Overnight cultures were subcultured at 1/100 in LB with appropriate antibiotics, with or without 1 mM H_2_O_2_ (VWR). Subcultures were incubated at 37°C with aeration. Aliquots were collected every hour for CFU determination. Results were expressed as percent survival calculated as [CFU(*t_n_*)/CFU(*t*_0_)] * 100 over time. Experiments were performed on at least three separate occasions. Statistical significance was determined using a Student’s *t* test.

### Hydrogen peroxide detection with Amplex red.

Overnight cultures were subcultured at 1/100 in LB broth with appropriate antibiotics containing 1 mM H_2_O_2_ (VWR). Uninoculated LB broth containing 1 mM H_2_O_2_ was used as a control and was treated in parallel with other samples. Subcultures were incubated at 37°C with aeration, and aliquots for hydrogen peroxide detection were collected every hour. Bacteria were collected by centrifugation at maximum speed (Eppendorf 5415D) for 3 min. Cleared supernatants were used for hydrogen peroxide detection using an Amplex red hydrogen peroxide/peroxidase kit according to the manufacturer’s protocol (Invitrogen). Hydrogen peroxide concentrations correlated with production of resorufin, and fluorescence was measured at 530/585 nm. Results were expressed as percent hydrogen peroxide degradation calculated as [fluorescence_530/585_(*t_n_*)/fluorescence_530/585_ (*t*_0_)] * 100 over time. Each experiment was performed on at least three separate occasions. Statistical significance was determined using a Student’s *t* test.

### Hydrogen peroxide sensitivity of mixed cultures in cross complementation studies.

Overnight cultures of the wild type and the siderophore or siderophore-processing mutant strain or the Δ*macAB* mutant and siderophore or siderophore-processing mutant strain were combined in a 1:1 ratio. The resulting mixed cultures were diluted 1/100 in LB broth supplemented with 1 mM H_2_O_2_ and incubated at 37°C with aeration. Aliquots were collected hourly, serially diluted, and plated on LB agar supplemented with appropriate antibiotics. Results were expressed as percent survival calculated as [CFU(*t_n_*)/CFU(*t*_0_)] * 100 over time. Each experiment was performed on at least three separate occasions.

### Siderophore extraction from the conditioned medium.

Overnight cultures of the wild type or the Δ*macAB* mutant were subcultured at 1/100 in 1 liter LB broth containing 0.5 mM H_2_O_2_ (VWR). Subcultures were grown at 37°C with aeration for 3 h. Bacteria were pelleted by centrifugation at 4,000 × *g* for 30 min, and conditioned medium was filtered to remove any remaining bacteria by the use of 0.2-μm-pore-size filters (Millipore). The resulting metabolite-containing medium was acidified to pH 2 with 10 N HCl. An additional liter of LB broth was acidified in a similar fashion as a media control for mock extraction.

Siderophores were extracted from the acidified conditioned media or mock LB control using a protocol previously described by Winkelmann et al. (77). Briefly, siderophores were extracted twice with an equal volume of ethyl acetate. The organic phases were combined and dried over anhydrous sodium sulfate. Solvents were evaporated using a Rotavapor (Büchi). Remaining residues were dissolved in 1 ml methanol. The siderophore concentration in these extracts was evaluated in Arnow assay (43).

### Arnow assay.

The presence of siderophore in the medium extracts was measured using Arnow assay (43). Fifty-microliter volumes of extract (from wild-type-conditioned and Δ*macAB* mutant-conditioned media) were mixed with an equal volume of 0.5 N HCl, followed by addition of nitrite-molybdate reagent (10 g sodium nitrate and 10 g sodium molybdate in 100 ml of H_2_O) and 1 N NaOH. In the presence of catecholate siderophores, this solution appears pink and has an absorption maximum at 510 nm. The concentration of siderophores in the extracts was calculated based on a calibration curve using enterobactin (Sigma) in a range from 0 to 100 μM.

### Cross-feeding assay.

An overnight culture of the indicator strain Δ*entA* (1 ml) was pelleted by centrifugation, washed twice with PBS, and resuspended in an equivalent volume of PBS. Melted LB agar containing 200 μM 2,2′-dipyridyl (DIP) was cooled to 55°C, inoculated with 25 μl of the Δ*entA* mutant, and used to pour plates. After agar plate solidification, agar plugs were removed from the plate using the wide end of a 200-μl pipette tip to create test wells. The resulting plates were used to detect the presence of siderophores in the siderophore extracts from the conditioned medium. Test wells were filled with 50 μl of extracts from the conditioned medium, and plates were incubated at 37°C. Positive (2.5 μM enterobactin [Sigma]) and negative (double-distilled water [ddH_2_O]) controls were included in each experiment. The Δ*entA* indicator strain cannot grow in the presence of the iron chelator DIP unless exogenous siderophores are present in the extracts added to the test well. The presence of a halo of growth around a test well indicated the presence of siderophores in the extract added to the given test well.

### HPLC analysis of siderophore extracts.

Siderophore extracts were separated on a reversed-phase HPLC column (Supelco) (Nucleosil C_18_, 5-μm pore size, 25 cm by 4 mm) using an acetonitrile gradient in water (6% to 40%) with 0.1% trifluoroacetic acid (TFA) with a flow rate of 1 ml/min and detection at 220 nm. Separation was performed on a Waters HPLC system. l-Tryptophan (100 μM) was added to each sample before injection into HPLC and served as a control for column performance.

An HPLC calibration kit containing enterobactin and its degradation products (enterobactin trimer [ENT-TRI], enterobactin-dimer [ENT-DIM], and enterobactin-monomer [ENT-MONO]) (EMC Microcollections, Germany) was used to determine the retention time for each siderophore derivative.

### HPLC fraction collection.

Fifty-microliter volumes of siderophore extracts from wild-type and Δ*macAB* mutant-conditioned media as well as the mock extract from LB broth were separated on HPLC as described above. Fractions were collected with the following retention times: 3.07 to 6.47 min (fraction 1 [negative control]); 6.48 to 10.07 min (fraction 2 [expected to contain ENT-MONO]); 10.08 to 13.47 min (fraction 3 [negative control]); 13.48 to 16.47 min (fraction 4 [expected to contain ENT-DIM]); 16.48 to 20.07 min (fraction 5 [expected to contain ENT-TRI]), and 20.08 to 24.07 (fraction 6 [expected to contain enterobactin]). Collected fractions were air-dried and reconstituted in 100 μl methanol. The concentration of siderophores was measured by Arnow assay as described above.

### Hydrogen peroxide sensitivity of the Δ*macAB* mutant in the presence of siderophore-containing HPLC fractions.

An overnight culture of the Δ*macAB* mutant grown in LB broth was diluted 1/100 in the fresh media supplemented with 1 mM H_2_O_2._ Fractions 1 to 6, with retention times corresponding to different siderophores or their metabolites as described above, were collected after HPLC and were added at a concentration of 2.5 μM (based on Arnow assay) to individual tubes containing the Δ*macAB* mutant and incubated at 37°C with aeration. A separate sample containing the Δ*macAB* mutant-inoculated LB broth with 1 mM H_2_O_2_ was incubated under similar conditions and served as a positive control. Aliquots were collected hourly, serially diluted, and plated on LB agar supplemented with chloramphenicol. Results were expressed as percent survival calculated as [CFU(*t_n_*)/CFU(*t*_0_)] * 100 over time. Each experiment was performed at least on three separate occasions.

### Hydrogen peroxide sensitivity of the Δ*macAB* mutant in the presence of purified enterobactin and its linearized products.

An overnight culture of the Δ*macAB* mutant grown in LB broth was diluted 1/100 in fresh medium supplemented with 1 mM H_2_O_2._ Enterobactin (Sigma), Ent-TRI, Ent-DIM, and Ent-MONO (all from EMC Microcollections, Germany) were added to individual tubes at 2.5 μM and incubated at 37°C with aeration. A separate tube containing the Δ*macAB* mutant-inoculated LB broth supplemented with 1 mM H_2_O_2_ was incubated under the same conditions and served as a positive control. Aliquots were collected hourly, serially diluted, and plated on LB agar supplemented with chloramphenicol. Results were expressed as percent survival calculated as [CFU(*t_n_*)/CFU(*t*_0_)] * 100 over time. Each experiment was performed on at least three separate occasions.
