# Supplementary figures and images for: Linearized Siderophore Products Secreted via MacAB Efflux Pump Protect Salmonella enterica Serovar Typhimurium from Oxidative Stress
Source: mBio. 2020 May 5;11(3):e00528-20. doi: 10.1128/mBio.00528-20 (PMC7403778; doi:10.1128/mBio.00528-20)

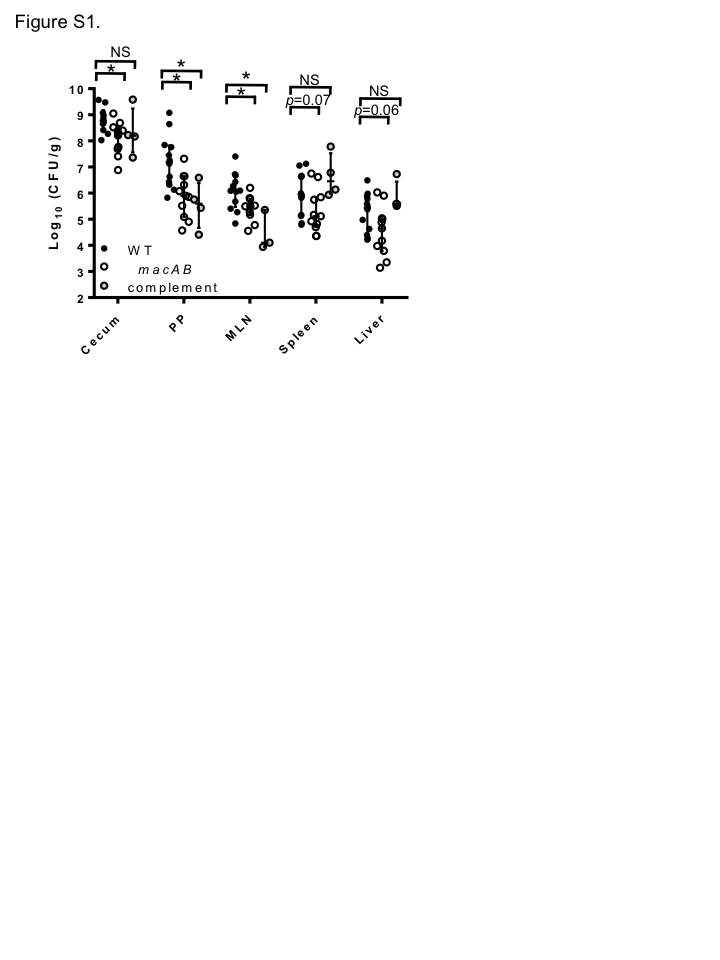

Supplement: FIG S1 [file mBio.00528-20-sf001.tif]

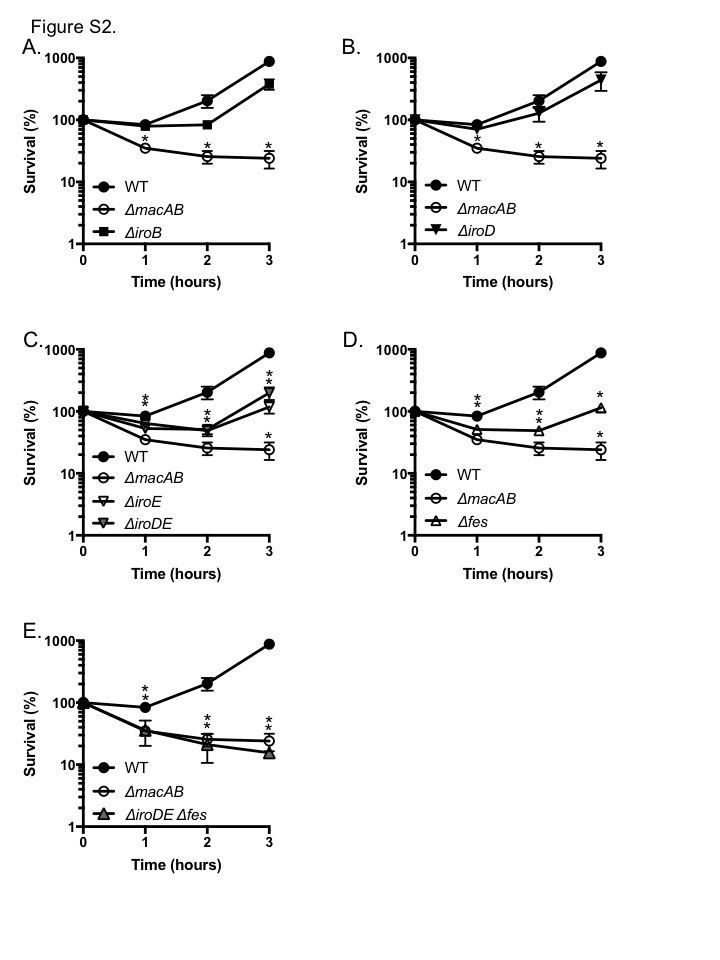

Supplement: FIG S2 [file mBio.00528-20-sf002.tif]

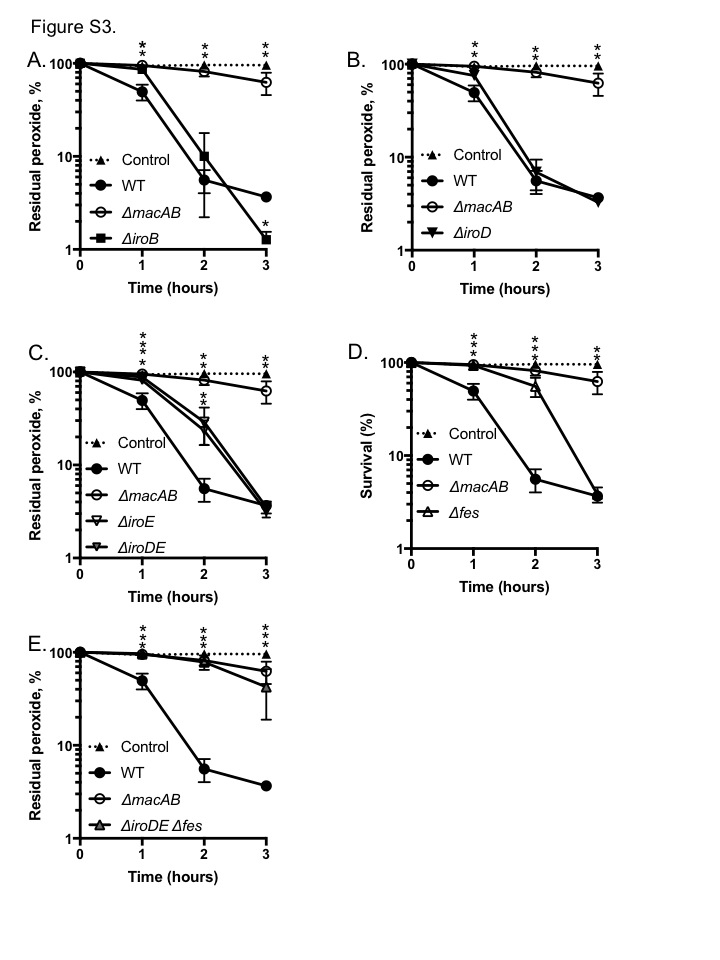

Supplement: FIG S3 [file mBio.00528-20-sf003.tif]

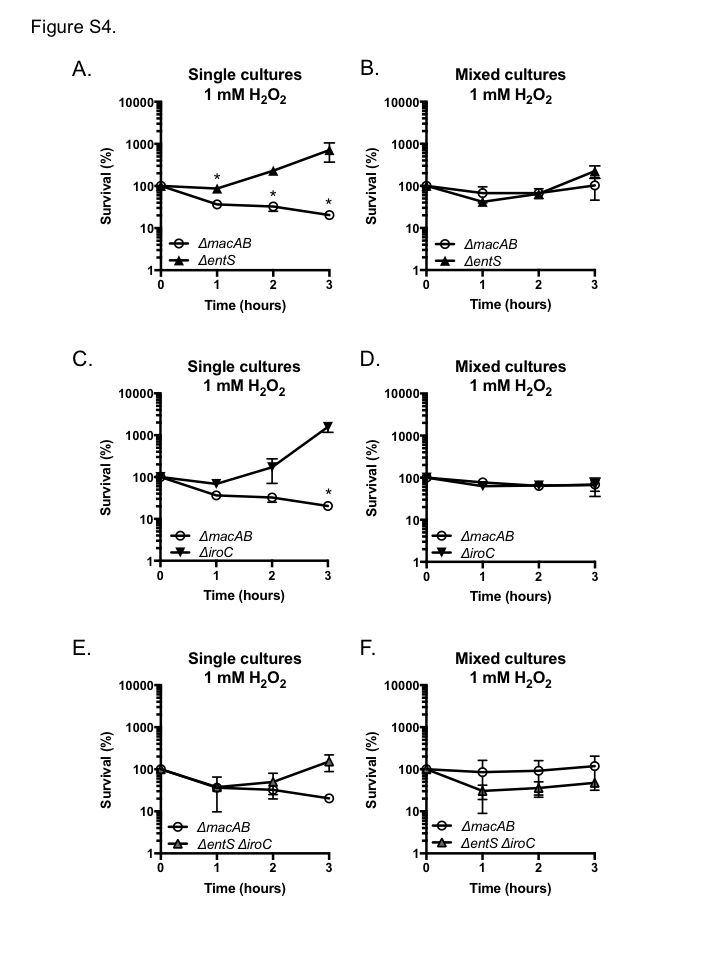

Supplement: FIG S4 [file mBio.00528-20-sf004.tif]

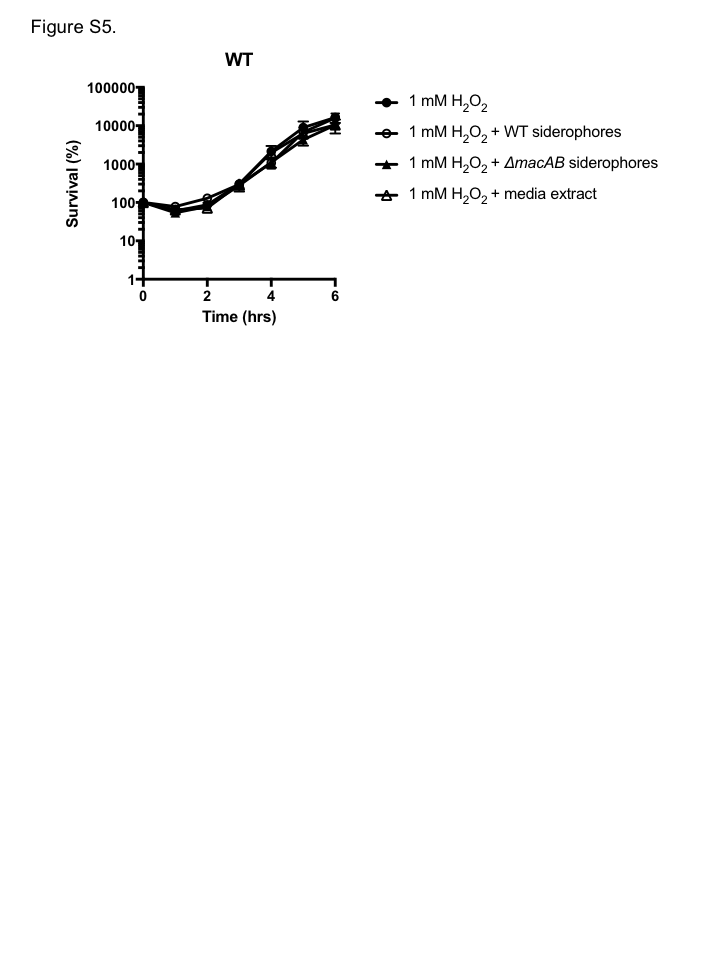

Supplement: FIG S5 [file mBio.00528-20-sf005.tif]

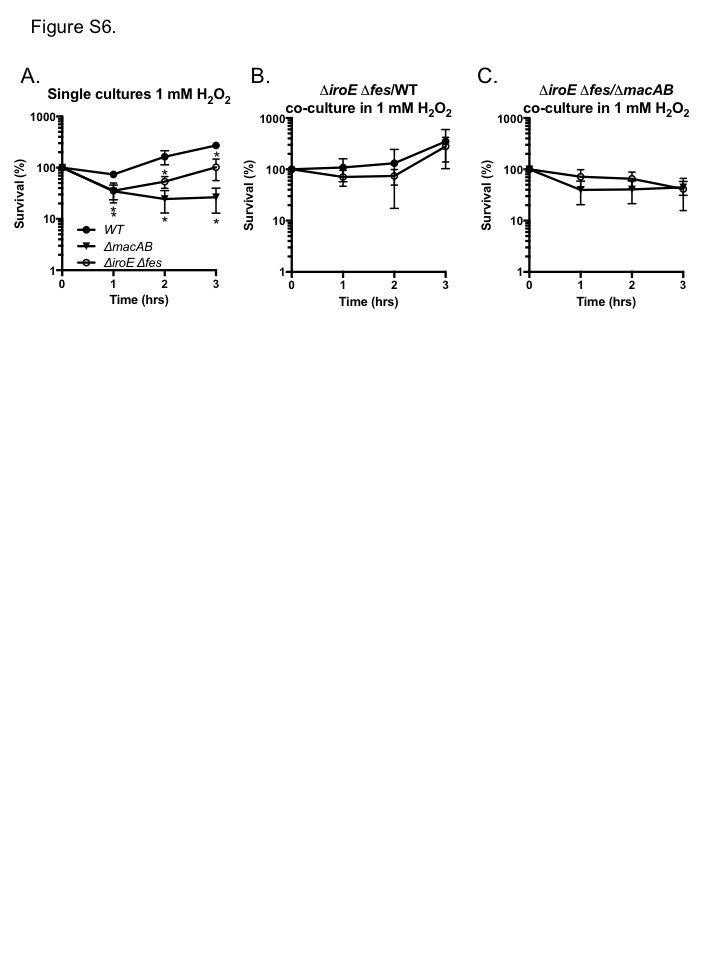

Supplement: FIG S6 [file mBio.00528-20-sf006.tif]
